# A Comprehensive Comparison of Different Selenium Supplements: Mitigation of Heat Stress and Exercise Fatigue-Induced Liver Injury

**DOI:** 10.3389/fnut.2022.917349

**Published:** 2022-05-12

**Authors:** Xinwei Chen, Jian Zhang, He Li, Wanlu Liu, Yu Xi, Xinqi Liu

**Affiliations:** National Soybean Processing Industry Technology Innovation Center, School of Food and Health, Beijing Technology and Business University, Beijing, China

**Keywords:** selenium-enriched soybean peptides, heat stress, exercise fatigue, heatstroke, oxidative damage, inflammation

## Abstract

This study aimed to compare the protective effects of different selenium supplements against heat stress and exercise fatigue-induced liver injury and to investigate the potential mechanisms of action. Selenium-enriched soybean protein (SePro), selenium-enriched soybean peptides (SePPs), and selenomethionine (SeMet) are organic selenium supplements in which selenium replaces the sulfur in their sulfur-containing amino acids. Common peptides (PPs) are obtained by enzymatic hydrolysis of soybean protein which was extracted from common soybean. The SePPs with higher hydrolysis degree and selenium retention were isolated *via* alkaline solubilization and acid precipitation and the enzymatic hydrolysis of alkaline protease, neutral protease, and papain. The results showed that SePPs could significantly increase the antioxidant levels in rats, inhibit lipid peroxidation, and reduce liver enzyme levels in rat serum, while the histological findings indicated that the inflammatory cell infiltration in the liver tissue was reduced, and new cells appeared after treatment with SePPs. Moreover, SePPs could increase glutathione (GSH) and GSH peroxidase (GSH-Px) in the liver, as well as protect the liver by regulating the NF-κB/IκB pathway, prevent interleukin 1β (IL-1β), interleukin 6 (IL-6), and tumor necrosis factor α (TNF-α) release in the liver. The SePPs displayed higher antioxidant and anti-inflammatory activity *in vivo* than SePro, SeMet, Sodium selenite (Na_2_SeO_3_), and PPs. Therefore, SePPs could be used as a priority selenium resource to develop heatstroke prevention products or nutritional supplements.

## Introduction

In recent years, with the general increase in the concept of health, more and more people are running, hiking, and doing other sports to promote their health. Meanwhile, some occupational groups (such as athletes, workers, etc.) require higher exercise intensity, which increases the incidence of exercise-related diseases. Therefore, sports health has drawn increasing attention during the past decades. Heatstroke is one of the common sports injury-related diseases, mainly caused by heat stress and exercise fatigue. Hyperthermic exercise is associated with severe fluid loss, which leads to the formation of reactive oxygen species (ROS) and affects the body’s redox balance (1). ROS can disrupt cell membranes and allow endotoxins and pathogens to leak into the human circulation, resulting in the destruction of the liver detoxification function and accompanying inflammatory response, eventually causing hepatocyte necrosis or liver tissue damage (2). Electrolyte or water supplementation alone has limited recovery from heatstroke induced inflammatory response and tissue damage. The most important treatment for patients with heatstroke is rapid and effective body cooling, while the endpoint of the cooling is controversial. The controversial lies in whether to stop cooling by targeting the patient’s core body temperature or behavioral performance (3). Consequently, developing functional foods or heatstroke preventive drinks has become a crucial and practical strategy to avoid heatstroke.

Selenium is an essential trace element for the body and a key component of the GSH-Px. GSH-Px is expressed in the cytoplasm and mitochondria, which can enhance the antioxidant capacity of the body and protect the structure and function of cells (4). The functions of selenium have been demonstrated previously, including detoxification, antioxidant and immune enhancement. It was reported that low selenium consumption was linked to various human disorders. Zhang et al. found that selenium deficiency disrupted the normal membrane structure of spleen cells, downregulated the expression levels of immune response-related genes, and activated T lymphocyte differentiation *via* the DUSP1/NF-κB pathway, affecting the immune response in the spleen and causing tissue damage (5). Selenium is an element that cannot be synthesized by the human body and needs to be consumed from food. Selenium supplements are classified as inorganic selenium (selenates and selenites) and organic selenium (selenocysteine [SeCys], selenomethionine [SeMet], selenoproteins and their hydrolysates) (6). Nevertheless, selenium is considered both beneficial and toxic depending on its form and amount ingested (7). The organic form of selenium is superior to the inorganic form in terms of bioavailability and toxicity for meeting human dietary requirements (8). Consequently, it is necessary to explore new selenium carriers to address the potential problems associated with selenium deficiency and selenium toxicity.

Soybean protein has a high nutritional value and contains a complete range of amino acids and eight essential amino acids (9). More importantly, soybean exhibits a high selenium enrichment capacity, with more than 75% of selenium in soybean bound to protein. Up to 82% of selenium is present in high molecular weight form, primarily in SeCys and SeMet (10). The bioavailability of selenium from soy protein can be as high as 86-96% (11). Previous results have shown that selenium-enriched soy protein hydrolysates are healthy nutrients. According to Liu et al., selenium biofortified soy peptides could reduce CCl_4_-induced liver fibrosis by increasing GSH-Px activity and GSH content, improving hepatocyte survival, inhibiting hepatic stellate cell activation, and improving antioxidant capacity (12). Zhang et al. observed that SePPs could regulate immunoglobulin (Ig)M, IgG, and IgA secretion, significantly increase splenic interleukin 2 (IL-2), interferon-gamma (IFN-γ), nitric oxide (NO), and cyclic guanosine monophosphate (cGMP) production, and IL-2, IFN-γ, and inducible NO synthase (iNOs) mRNA expression, as well as enhance host-specific and non-specific immunity *via* a cyclophosphamide-induced immunosuppression mice model (13). The beneficial effects of selenium-enriched peptides on health were confirmed previously, whereas, the protective mechanism of selenium-enriched peptides against heatstroke-induced liver injury is still unclear. Meanwhile few studies attempted to compare the effects of different selenium supplements.

This study, isolated SePro from naturally grown selenium-enriched soybeans, which were enzymatically separated to produce SePPs. A modeling approach of rats swimming in hot water under heat stress and exercise fatigue-induced heatstroke was used to investigate the protective effect of SePPs against sports injury and the possible mechanisms of action. The impact of different forms of selenium supplements was compared comprehensively. It was predicted that SePPs would prevent heatstroke and increase exercise tolerance by reducing oxidative stress and regulating inflammatory responses. This study provides a basis for preventing sports diseases and also provides new ideas for selenium nutrition supplementation.

## Materials and Methods

### Materials and Chemicals

Selenium-enriched soybeans were purchased from Enshi Se-Run Health Tech Development Co., Ltd. (Enshi city, China), while L-SeMet was obtained from Shanghai Macklin Biochemical Co., Ltd. Na_2_SeO_3_ was purchased from Sigma Chemical Co. (St. Louis, MO, United States). Xi’an Ruidi Biotechnology Co., Ltd., supplied the honeysuckle extract. The alanine aminotransferase (ALT), aspartate aminotransferase (AST), catalase (CAT), superoxide dismutase (SOD), malondialdehyde (MDA), GSH, and GSH-Px kits were obtained from Nanjing Jiancheng Bioengineering Institute. The IL-1β, IL-6, TNF-α, GSH, and GSH-Px ELISA kits were acquired from Jiangsu Meibiao Biotechnology Co., Ltd., while the BCA protein concentration assay kit was purchased from Beijing Solarbio Science & Technology Co., Ltd. The skim milk powder was supplied by Becton, Dickinson, and Company. Furthermore, the PVDF membrane, TBST, NF-κB p65 rabbit monoclonal antibody, phospho-IκBα (Ser32/36) rabbit polyclonal antibody, α-Tubulin rabbit polyclonal antibody, horseradish peroxidase-labeled goat anti-rabbit IgG (H + L), Western secondary antibody dilution, and BeyoECL Star (extra super-sensitive enhanced chemiluminescence kit) were purchased from Beyotime Biotechnology.

### Preparation of SePPs

**FIGURE 1:**
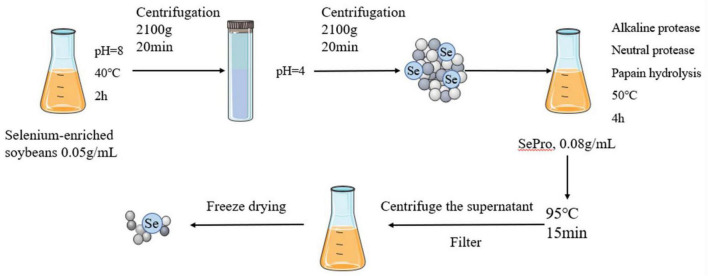
Preparation of SePPs.

The SePPs was prepared as described in a previous study (14). The selenium-enriched soybeans were crushed, defatted, and dried to obtain a powder, after which the SePro was extracted *via* alkaline solubilization and acid deposition. The preparation process is shown in Figure 1. Alkaline solubilization was performed at 40°C for 2 h at a 1:20 mater-liquid ratio and pH = 8. The SePro was obtained *via* centrifugation at 2,100 g for 20 min, after which the supernatant was adjusted to pH 4.0 and centrifuged at 2,100 g for 20 min. Subsequently, the SePro was dissolved in water to obtain a SePro solution with a mass fraction of 8%, using alkaline protease, neutral protease, and papain. Enzymatic digestion occurred at 50°C for 4 h a ratio of 2:*1*:1. Finally, the enzymatic digest was heated at 95°C for 15 min to inactivate the protease, to ensure that the degree of hydrolysis reached 68%. After centrifugation, the supernatant was collected and filtered through a 0.45 μm microporous membrane. SePPs were obtained after freeze-drying.

Protein content, selenium content and amino acid composition were determined as described in previous reports (14). The protein content in the SePro, SePPs, and common soy peptides (PPs) was assessed using the Kjeldahl method (Kjeltec 8000, FOSS analysis A/S) at a conversion factor setting of 6.25. The total selenium content in the solid SePro, SePPs and PPs powders was measured using hydride generation-atomic fluorescence spectrometry (LC-AFS6500, Beijing Haiguang Instruments Co., Ltd.). The amino acid composition of SePro, SePPs, and PPs was assessed using an amino acid analyzer (Biochrom 30 amino acid analyzer; Biochrom Ltd.) and a Na cation exchange column (8 mm, 4.6 × 200 mm), which was purchased from the Waters Corporation. The amino acids were derivatized with ninhydrin reagent after they passed through the exchange column. The absorbance of the subsequent compounds was measured at 440 nm (proline [Pro]) and 570 nm (all other amino acids). The molecular weight distribution of SePPs was determined following a previously reported method (13). Standard molecular weight samples of aprotinin (6500 Da), bacitracin (1422 Da), Gly-Gly-Tyr-Arg (451 Da), and reduced GSH (307 Da) were passed through a 0.22 μm filter and deposited in a Superdex 200 10/300 GL column. The chromatographic analysis was performed using an ÄKTA pure system (AKTA pure 25, Cytiva). The phosphate buffer (PBS, concentration 0.05 M, pH = 7) mobile phase was eluted at a 0.5 ml/min flow rate and detected at 220 nm.

### Animals and Diet

Eighty male Sprague-Dawley (SD) rats (6 weeks old, weight 170 ± 10 g) were purchased from Beijing Vital River Laboratory Animal Technology Co., Ltd., license No. SCXK (Beijing) 2016-0006. The rats received a standard diet and were provided free access to water. They were kept at a temperature of 22 ± 2°C, a humidity of 50 ± 10%, and a 12 h light/12 h dark cycle. The rats were acclimatized for one week before the experiment. The experimental protocol was approved by the Institutional Animal Care and Use Committee at the Pony Testing International Group Co., Ltd. (PONY-2021-FL-15).

### Establishing the Heatstroke Rat Model

The 80 male SD rats were randomly divided into eight groups (*n* = 10): control group, model group, positive control (PC) honeysuckle group (15), SePro group, SePPs group, PPs group, SeMet group, and Na_2_SeO_3_ group. All rats were administered *via* gavage, while the control and model groups were given equal volumes of deionized water, in which the selenium content 18 μg/kg in selenium-containing groups was equal, equivalent to 3.57 μg/kg in adults (Reference Chinese Nutrition Association: The recommended adult daily intake of selenium is 50-250 μg, 200 μg was selected for conversion). The protein composition of the PPs group was the same as that of SePPs group, and the rats were gavaged once daily for 7 d. The last gavage was administered after a 12-h fasting period. After 10 min of gavage, the rats in the control group were kept at room temperature (22 ± 2°C), while the rats in the model group were placed in hot water to swim at 40 ± 2°C to observe their status. When the activity of the rats decreased, the hot water near them was agitated with a wooden stick to keep them moving continuously. The rats were removed immediately when they started to sink, and the time was recorded. The remaining groups of rats swam in hot water for the same duration as the model group. After completing the swimming process, the rats were anesthetized with 3% sodium pentobarbital and dissected to collect the material. Blood was taken from the abdominal aorta, serum was separated for blood indicator analysis, and liver tissue was collected for histomorphometric analysis, while the rest was flash-frozen in liquid nitrogen and stored at −80°C for subsequent analysis.

### Determination of the Oxidative Stress Indicators in the Serum

The SOD, CAT, GSH-Px, MDA, and GSH levels in serum were measured using commercial kits according to the instructions of the manufacturer.

### Determination of Serum Liver Enzyme Levels

The ALT and AST viability in serum were measured using commercial kits according to the instructions of the manufacturer. The optical density (OD) values of the solutions were measured at 510 nm using an automated microplate reader (Infinite 200 Pro Nanoquant, Tecan).

### Determination of the GSH and GSH-Px Levels in the Liver Tissues

Tissue samples were collected and rinsed with saline. The filter paper was blotted to dry the surrounding water, after which the tissues were cut, weighed, and placed on ice with a delay of 5 s after every 5 s of grinding. The grinding process was repeated five times for tissue homogenization, and the tissue homogenate used PBS (pH = 7.2-7.4, concentration of 0.01 mol/L) with a homogenization ratio of 10%. The supernatant was collected for analysis after centrifugation at 2296 g for 15 min. The GSH and GSH-Px levels in liver tissue were measured using a commercial rat ELISA quantification kit according to the instructions of the manufacturer. The OD values of the solutions were measured at 450 nm using an automated microplate reader (Infinite 200 Pro Nanoquant, Tecan).

### Histological Analysis

Some of the liver tissue was immediately fixed in formalin for 48 h, embedded in paraffin, stained with hematoxylin and eosin, and connected to a microscope for observation and morphological analysis, which was used to assess the degree of liver tissue damage.

### Determination of the Inflammatory Factors in Liver Tissue

The liver tissue was pre-treated as described in Section 2.7. The IL-1β, IL-6, and TNF-α concentrations in the liver tissue were measured using commercial rat ELISA quantification kits according to the instructions of the manufacturer. The OD values of the solutions were measured at 450 nm using an automated microplate reader (Infinite 200 Pro Nanoquant, Tecan).

### Western Blot

The liver samples were added to the lysate (RIPA) with phosphatase inhibitors, placed on ice and ground, and delayed for 5 s after every 5 s of grinding. The grinding process was repeated five times, after which the extracts were centrifuged for 4 min at 12000 g. The protein concentration was determined by adding diluent to the BCA supernatant to unify the protein concentrations of all the samples. Then 5 × loading buffer was added and placed in a 100°C water bath. The mixture was heated for 5 min for denaturing, cooled, and centrifuged at 9184 g for 2 min, after which the supernatant was collected for determination. The protein was separated in 10% SDS-PAGE and transferred to a PVDF membrane. After blocking with 5% skim milk, the primary antibody (NF-κB p65 rabbit monoclonal antibody, phospho-IκBα (Ser32/36) rabbit polyclonal antibody, 1:1000) was incubated overnight at 4°C, after which the primary antibody was washed off. The secondary antibody [anti-rabbit IgG(H + L) 1:1000] was incubated for 1 h at room temperature, and protein expression was normalized with α-Tubulin *via* ECL chemiluminescence. Band intensities were measured using Image J software.

### Statistical Analysis

The data were expressed as mean ± standard deviation (SD). The results were analyzed *via* one-way analysis of variance (ANOVA) and Tukey’s method using SPSS 23 software. A P value less than 0.05 indicated statistical significance.

## Results

### Characterization of SePPs

The protein contents of the SePro, SePPs, and PPs were 89.36 ± 0.24%, 84.46 ± 0.03%, and 88.98 ± 0.15%, respectively, while the total selenium contents in the solid SePro, SePPs, and PPs powders were 44.00 ± 0.95 mg/kg, 26.00 ± 0.7 mg/kg and 0.06 ± 0.001 mg/kg, respectively. During alkaline solubilization and acid deposition, the proteins were separated *via* acid-base washing and dialyzed to remove the small molecules (including inorganic selenium not attached to the proteins), leaving only organic selenium in SePro and SePPs. Table 1 showed the amino acid compositions of the SePro, SePPs, and PPs. SePro, SePPs, and PPs contained a comprehensive variety of amino acids, including eight essential amino acids, as well as aliphatic, aromatic, and basic amino acids.

**TABLE 1:** The amino acid compositions (%) in SePro, SePPs, and PPs.

	SePro (%)	SePPs (%)	PPs (%)
Asp	10.63 ± 0.02	12.37 ± 0.17	13.00 ± 0.12
Thr	3.33 ± 0.23	4.1 ± 0.07	3.70 ± 0.08
Ser	5.64 ± 0.12	6.4 ± 0.07	6.17 ± 0.03
Glu	17.79 ± 0.31	21.67 ± 0.16	24.78 ± 0.53
Pro	5.12 ± 0.17	4.17 ± 0.36	5.38 ± 0.33
Gly	7.37 ± 0.17	7.82 ± 0.09	4.15 ± 0.06
Ala	6.96 ± 0.04	7.05 ± 0.08	3.48 ± 0.02
Cys	0.29 ± 0.05	0.19 ± 0.01	1.91 ± 0.06
Val	6.03 ± 0.03	4.11 ± 0.06	2.24 ± 0.01
Met	1.05 ± 0.02	1.9 ± 0.09	0.78 ± 0.04
Ile	5.98 ± 0.26	6.66 ± 0.18	2.47 ± 0.01
Leu	8.34 ± 0.34	6.01 ± 0.16	7.17 ± 0.19
Tyr	3.19 ± 0.21	2.09 ± 0.02	3.36 ± 0.07
Phe	4.66 ± 0.22	3.15 ± 0.17	5.38 ± 0.14
His	2.24 ± 0.05	2.48 ± 0.04	2.24 ± 0.01
Lys	5.75 ± 0.13	5.21 ± 0.31	6.17 ± 0.27
Arg	5.63 ± 0.04	4.64 ± 0.03	7.62 ± 0.28

As shown in Table 2, more than 90% of the components of SePPs and PPs have molecular weights less than 3000 Da. The molecular weight of peptides represents a vital influencing factor of their functional activity, while bioactive peptides are typically a mixture of protein hydrolysis products and low molecular weight peptides.

**TABLE 2:** Molecular mass distribution of PPs and SePPs.

Molecular weight (Da)	The content of PPs (%)	The content of SePPs (%)
>3000	4.64 ± 0.001	5.41 ± 0.001
1500-3000	7.66 ± 0.001	7.52 ± 0.001
1000-1500	10.94 ± 0.002	10.61 ± 0.001
500-1000	20.17 ± 0.003	22.99 ± 0.003
<500	56.58 ± 0.006	53.47 ± 0.005

### Effects of Different Selenium Supplements on the Oxidative Damage in the Heatstroke Rats *in vivo*

**FIGURE 2:**
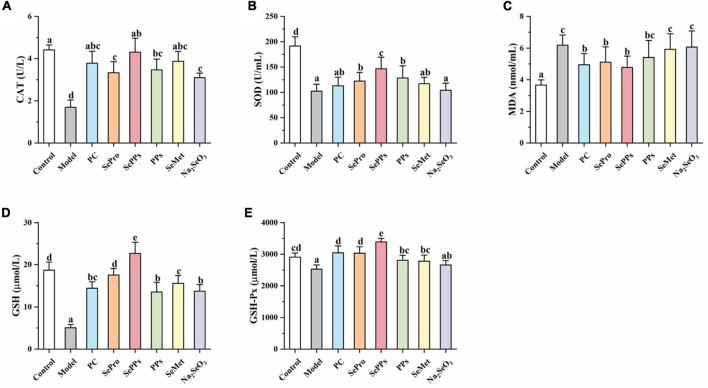
Effects of different selenium supplements on CAT **(A)**, SOD **(B)**, MDA **(C)**, GSH **(D)**, and GSH-Px **(E)** in rat serum. Values are expressed as the mean ± SD, results marked with the same letters were not significantly different (*p* > 0.05).

When the rats were removed from the hot water, their eyes were dull and bulging, while most were not moving, indicating the occurrence of heatstroke. The CAT, SOD, GSH-Px, MDA, and GSH levels in the serum were measured to evaluate the ameliorative effect of SePPs on oxidative damage *in vivo* in the heat stress and exercise fatigue-induced heatstroke rats. As shown in Figure 2. Heatstroke significantly decreased (*p* < 0.05) the CAT, SOD, GSH-Px, and GSH content in the serum of the rats in the model group by 61, 46, 13, and 73%, respectively, while the MDA content was 170% higher than that in the control group, suggesting stronger oxidative stress in the rats after heatstroke. Compared with the model group, significant differences were found in the CAT, SOD, MDA, GSH, and GSH-Px levels in serum between the SePro and SePPs groups (*p* < 0.05), while no statistical difference was observed in the MDA content levels in the serum between the PPs and model groups (*p* > 0.05). Additionally, there were no statistical differences between the SOD activity and MDA content in the serum of the rats in the SeMet and model groups (*p* > 0.05), while the serum SOD, GSH-Px activities, and MDA content of the rats in the Na_2_SeO_3_ group were not statistically different from those in the model group (*p* > 0.05). Our results indicated that SePPs could improve oxidative damage due to heatstroke by increasing the antioxidant enzyme activity and inhibiting lipid peroxidation. Furthermore, its ability to increase antioxidant enzyme activity in the body was higher than that of SePro, PPs, SeMet, and Na_2_SeO_3_, while the degree of lipid peroxidation inhibition was not statistically different from that of SePro (*p* > 0.05).

### Effect of Different Selenium Supplements on the Serum Liver Enzyme Levels in Heatstroke Rats

**FIGURE 3:**
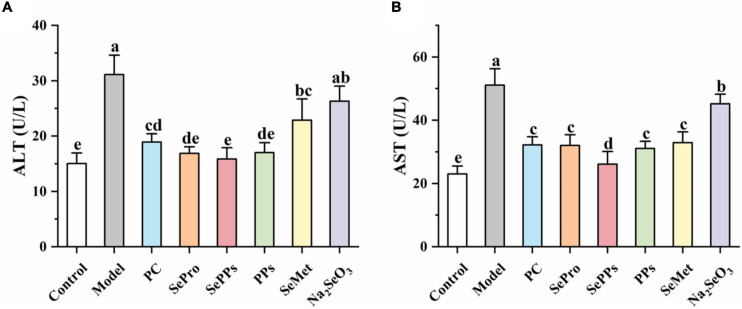
Effects of different selenium supplements on ALT **(A)** and AST **(B)** in rat serum. Values are expressed as the mean ± SD, results marked with the same letters were not significantly different (*p* > 0.05).

The ALT and AST levels in the serum of the rats were determined to assess the impact of heat stress and exercise fatigue on the liver. It has been shown in Figure 3 that the liver enzyme levels in the serum of the rats in the model group were significantly higher (*p* < 0.05), which were 2.0 and 2.2 times higher than those in the control group. The data indicate the impaired liver function after heatstroke. The SePro, SePPs, PPs, and SeMet groups displayed significantly lower (*p* < 0.05) liver enzyme blood levels than the model group, while the serum ALT in the Na_2_SeO_3_ group did not differ considerably from the model group (*p* > 0.05). SePro, SePPs, and PPs were more effective in reducing the serum ALT levels than SeMet and Na_2_SeO_3_, while SePPs were the most effective in lowering serum AST among the other treatment groups. Overall, SePPs were superior to other supplements in preventing liver damage.

### Effect of Different Selenium Supplements on the GSH and GSH-Px Content in the Liver Tissue of Heatstroke Rats

**FIGURE 4:**
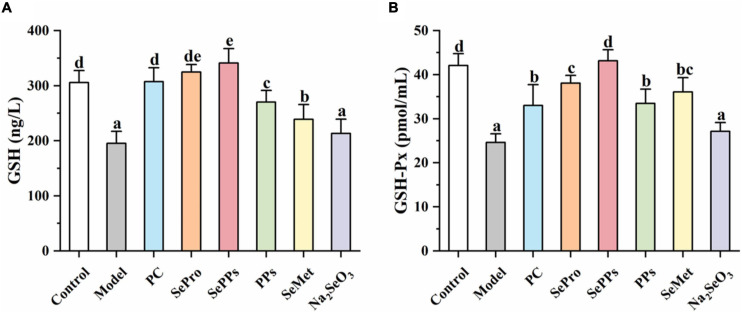
Effects of different selenium supplements on GSH **(A)** and GSH-Px **(B)** in rat liver. Values are expressed as the mean ± SD, results marked with the same letters were not significantly different (*p* > 0.05).

Selenium is a vital component of GSH-Px and is closely related to the GSH system. Therefore, the GSH and GSH-Px content in the liver tissues were determined further. The results are shown in Figure 4. The GSH and GSH-Px content in rat livers was considerably reduced (*p* < 0.05) after heatstroke, which was 64 and 59% of the control, respectively. Supplementation with SePro, SePPs, PPs, and SeMet before heat stress and exercise improved the decrease in GSH and GSH-Px content (*p* < 0.05). However, Na_2_SeO_3_ supplementation did not significantly augment the GSH and GSH-Px levels in the liver (*p* > 0.05). The GSH contents in the SePPs group were higher than in the control group.

### Histopathological Analysis of the Liver

**FIGURE 5:**
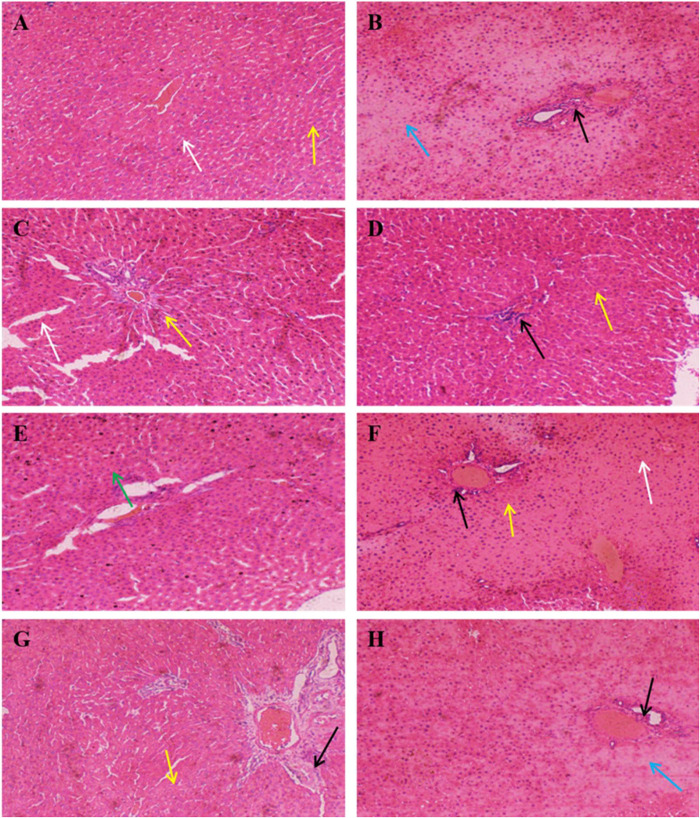
Histopathology of liver (H&E, × 100). Yellow arrows indicate hepatic sinusoids; blue arrows indicate necrosis of hepatocytes and disappearance of nuclei consolidation and lysis; white arrows indicate hepatic sinusoidal macrophages; black arrows indicate inflammatory cells; green arrows indicate neoplastic cells. **(A)** Control group, **(B)** Model group, **(C)** PC group, **(D)** SePro group, **(E)** SePPs group, **(F)** PPs group, **(G)** SeMet group, **(H)** Na_2_SeO_3_ group.

The results of histopathological analysis were presented in Figure 5. The overall structure of the liver tissue in the control group was normal, with a complete hepatocyte structure, and a clearly visible central vein, while the hepatic sinusoids were arranged radially along the central vein, as shown by the yellow arrows. The white arrows indicated the hepatic sinusoidal macrophages, and no obvious inflammatory cell infiltration was evident in the tissue. In contrast, the overall structure of the liver tissue in the model group was abnormal, with a loose hepatocyte structure, hepatocyte necrosis, and the disappearance of nucleus fixation and lysis, as shown by the blue arrows, while the black arrows indicated the presence of inflammatory cell infiltration in the tissue. The addition of SePro, SePPs, PPs, and SeMet improved liver tissue damage to varying degrees, while Na_2_SeO_3_ supplementation did not facilitate significant improvement. The overall structure of the liver tissue was mildly abnormal in the SePro, PPs, and SeMet groups, with visible inflammatory cell infiltration, as shown by the black arrows. Moreover, significantly dilated hepatic sinusoids were evident in the SePro and SeMet groups, as shown by the yellow arrows. Notably, the SePPs group displayed a complete hepatocyte structure, and a clearly visible central vein. The tissue did not show obvious sparing edema necrosis of the hepatocytes, and no noticeable inflammatory cell infiltration was apparent. Additionally, several new hepatocytes were visible, as shown by the green arrows in Figure 5E.

### Effect of Different Selenium Supplements on the Inflammatory Factor Concentration in the Liver Tissue of Heatstroke Rats

**FIGURE 6:**
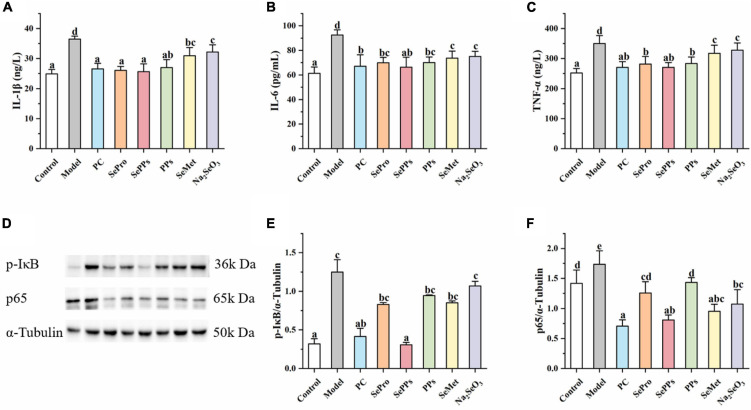
Effects of different selenium supplements on IL-1β **(A)**, IL-6 **(B)**, TNF-α **(C)**, Western blot analysis **(D)**, p-IκB **(E)**, and p65 **(F)** in rat liver. Values are expressed as the mean ± SD, results marked with the same letters were not significantly different (*p* > 0.05).

Various physiological responses, such as the inflammatory response, occur *in vivo* in heat stress conditions. IL-1β, IL-6, and TNF-α are regarded as important indicators during heatstroke studies. As shown in Figures 6A-C, the IL-1β, IL-6, and TNF-α levels in the livers of the rats in the model group were 1.5, 1.5, and 1.4 times of in the control group (*p* < 0.05) after exposure to heat stress and exercise, indicating a stronger inflammatory response in the liver tissue. Compared to the model group, IL-1β, IL-6, and TNF-α were substantially lower in the SePro, SePPs, PPs, SeMet, and Na_2_SeO_3_ groups (*p* < 0.05). It seemed that selenium and nutritional protein supplementation suppressed these substances after heat stress and exercise, while SePro, SePPs, and PPs were more effective than SeMet and Na_2_SeO_3_.

### Effect of Different Selenium Supplements on the Expression of Related Proteins in the NF-κB/IκB Signaling Pathway of Heatstroke Rat Liver Tissues

The NF-κB p65 and p-IκB protein levels in the different treatment groups were measured to investigate the role of the NF-κB/IκB signaling pathway in heatstroke. As shown in Figures 6D-F, the protein expressions of p-IκB and NF-κB p65 were upregulated in the livers of rats after heat stress and exercise. The SePPs treatments significantly reduced p-IκB and NF-κB p65 protein expressions compared with the model group (*p* < 0.05). The protein expression of p-IκB in SePro, PPs, SeMet, and Na_2_SeO_3_ treatment groups were not significantly different from that of the model group (*p* > 0.05). The results indicated that supplementation with selenium and nutritional proteins could improve the inflammatory response by inhibiting the expressions of p-IκB and NF-κB p65 protein *in vivo*. SePPs were the most effective of the selenium supplements, variation was evident in the regulation of different transcription factors, while the regulation of p65 protein expression was superior to that of p-IκB.

## Discussion

The morbidity and mortality of sports diseases have attracted increasing attention in recent years. However, in the context of dramatic global warming, there is no specific and effective treatment strategy for heat stress and exercise fatigue-induced heatstroke. Therefore, it is essential to study on the prevention of heatstroke. The primary aim of this study was to investigate the protective effect and potential action mechanism of SePPs in heat stress and exercise fatigue-induced heatstroke rats. This study focuses on the liver since it is more sensitive to heat stress, while liver injury and dysfunction represent the most common and often fatal pathological changes in almost all cases of heat stress (16). Additionally, the liver is the central organ for selenium metabolism. Selenium ingested *via* food is absorbed in the intestine and transported to the liver for metabolism, from where it is transported and distributed to other tissues in the body (17). This study selected honeysuckle as a PC due to its antioxidant and anti-inflammatory effect. Using Freund’s adjuvant-induced arthritis rat model, Wu et al. found that honeysuckle extract inhibited the production of pro-inflammatory factors (TNF-α, IL-1β, IL-6, NO/iNOS, and cyclooxygenase-2 [COX-2]) and increased the activity of antioxidant enzymes (SOD, GSH-Px, and heme oxygenase-1 [HO-1]), attenuating arthritis symptoms (18). Honeysuckle also exhibits hepatoprotective effects (19).

Heatstroke causes oxidative damage, which is related to the pathophysiology of various disorders (20), including respiratory muscle dysfunction in heart failure (21) and skeletal muscle exhaustion *in vitro* (22). CAT, SOD, and GSH-Px are key components of the antioxidant enzyme system in living organisms and play a significant role in maintaining oxidative and antioxidant equilibrium in the body. GSH helps maintain normal immune system function, displays antioxidant effects, and integrates detoxification. MDA is the end product of lipid peroxidation and induces the cross-linking polymerization of proteins, nucleic acids, and other biological macromolecules. This study showed a decrease in antioxidant enzyme activity and an increase in lipid peroxidation after heat stress and exercise, consequently causing oxidative stress. Zhang et al. found that selenium-containing soybean antioxidant peptides inhibited D-galactose-induced liver injury and oxidative brain damage *via* the MAPK/NF-κB pathway, and played a key role in reducing organic oxidative damage during inflammation and aging. They compared the antioxidant activity of inorganic selenium, organic selenium, and selenium-free peptides and found that the effect of the selenium-enriched peptide treatment group was more significant (23). Consistent with these findings, this study revealed that PPs exhibited an ameliorative effect on the oxidative damage caused by heat stress and exercise fatigue. However, the effect of the SePPs treatment group was superior to PPs and other forms of selenium supplementation, indicating that both selenium and peptides play a vital role in antioxidant activity. Many studies have reported the antioxidant effect of selenium (24), while its bioavailability is essential for the strength of functional expression. We improved selenium bioavailability *via* selenium biofortification with soy protein, soy peptides, and methionine as carriers, respectively. Protein stability and transport pathways *in vivo* determine the true efficacy of functional applications. The oral bioavailability of such macromolecules is generally low due to poor membrane permeability. Proteins are typically denatured by pepsin and produce a mixture of peptides with molecular weights > 10 kDa. These gastric digestion products are further hydrolyzed by pancreatic enzymes, indicating that they exhibit low stability in the gastrointestinal tract and are susceptible to degradation by multiple digestive enzymes. Therefore, they are difficult to be absorbed by the human body completely (25). SeMet is absorbed through the intestinal methionine transporter after ingestion, following the active amino acid absorption pathway. SeMet and Met can share the same Na^+^-dependent, carrier-mediated transport mechanism (26), which is also an important way for multiple amino acids to be absorbed by the intestine. This may decrease SeMet absorption efficiency due to the structural similarity of individual amino acids and mutual competition. The higher bioavailability exhibited by SePPs may be due to their higher stability and membrane permeability, as well as their rapid *in vivo* transport and less saturable carriers. Furthermore, the results indicated that different forms of selenium exhibit varying bioavailability, while that of organic selenium was significantly higher than inorganic selenium. The body can absorb inorganic and organic selenium *via* the digestive tract, particularly the small intestine. However, a large amount of inorganic selenium is lost *via* urine as selenite and selenate before further metabolism, resulting in the bioavailability of selenium as selenite and selenate at levels lower than organic selenium (27). In line with previous findings, acute heat exposure increased lipid peroxidation and protein oxidation in rat plasma, ultimately resulting in oxidative damage to the body (28). As a metabolic crossroads, the liver is vulnerable to heat stress and exercise state, disturbing the metabolic equilibrium of the liver (29). Moreover, the liver is high in selenium, accounting for 29% of the total selenium in rats (27). It is the first organ encountered after selenium absorption in the small intestine and plays a crucial role in selenium metabolism (30). Consequently, more research regarding heat stress and exercise fatigue-induced liver injury was conducted.

The liver secretes several endogenous and exogenous compounds during detoxification. ALT and AST are hepatic enzymes, the levels of which can be used as key indicators of liver function. Our results showed that heatstroke compromised the integrity of hepatocyte membranes, allowing aminotransferases to escape into the serum from the cytoplasm. Generally, the increase in the degree of ALT and AST in serum reflects the degree of hepatocyte injury. ALT is mainly present in the cytoplasm of hepatocytes, while AST is primarily distributed in the cytoplasm and mitochondria of hepatocytes. Elevated ALT or AST level in the blood suggested hepatocyte membrane injury and high membrane permeability or organelle damage, respectively (31). Guo et al. found that selenium biofortified maize peptide exerted a substantial ameliorative impact on the high ALT and AST caused by concanavalin A (32). Similarly, the present study revealed that advanced supplementation with different forms of selenium reduced liver damages induced by heat stress and exercise fatigue and PPs displayed hepatoprotective benefits.

As protein degradation products, peptides play an essential role in the development of various liver disorders. In the investigation of Lin et al., marine collagen peptides inhibited early alcoholic liver injury in female rats by improving oxidative stress and lipid metabolism (33). Lv et al. observed that maize peptides significantly reduced MDA, NO, hydroxyproline (HYP), transforming growth factor-β1 (TGF-β1) levels and lactate dehydrogenase activity in the liver and substantially increased the SOD level. The action mechanism of maize peptides might be related to their antioxidant activity in hepatocytes and TGF-β1 secretion inhibition (34). Recently, SePPs were found to exhibit better immunomodulatory effects than PPs in a mouse model involving cyclophosphamide-induced immunosuppression (13). In our study, SePPs had a more significant impact on the GSH system than PPs. The serum indicator results demonstrated that SePPs were most effective in regulating GSH and GSH-Px. Consequently, the GSH and GSH-Px levels in the liver were investigated further, showing that the GSH system was altered after heat stress and exercise fatigue. Using a broiler model, Yang et al. found that acute heat exposure could inhibit the mitochondrial respiratory chain activity, resulting in excessive ROS production and eventually oxidative stress (35). Excessive ROS production overwhelms cellular antioxidant defenses, causing damage to various cellular components (36). The liver is responsible for neutralizing excess ROS by disrupting the mitochondrial membrane proton gradient, causing oxidative damage by breaking the loops of enzymes, cellular lipids, and mitochondrial membranes in the liver. Furthermore, ROS can also act as signaling molecules, affecting the expression of related genes, requiring regulation by the antioxidant system (37–39), which includes GSH and GSH-Px. Similar to the results obtained by Liu et al. (12), the GSH content and GSH-Px activity in the serum and liver of the rats in the SePPs group were higher than those in both PPs group and control group, which may be attributed to the unique relationship between selenium and the GSH system. The increased SePPs on GSH-Px activity may be related to the presence of SeCys peptides in SePPs. Since SeCys represents the active GSH-Px center (40), SePPs can be used as a synthetic GSH-Px and increase its activity, converting more GSH to oxidized GSH (GSSG). Then, catalyzed by GSH reductase, GSSG can be reduced to GSH again by nicotinamide adenine dinucleotide phosphate (NADPH) (41). Throughout the redox cycle, higher GSH-Px activity stimulated the involvement of GSH in the reaction, resulting in higher GSH levels in the SePPs-treated group. This indicated that SePPs more successfully regulated the GSH system in the liver than PPs, reducing oxidative damage. In addition, the GSH content affected the TNF-α sensitivity to hepatocyte destruction. The cell death process includes the mitochondrial release of cytochrome C, while a decrease in the cytoplasmic GSH content is necessary for cytochrome C to promote apoptosis (42). According to Matsumaru et al., GSH depletion in the body enhanced TNF-α sensitivity to induce apoptosis in primary murine hepatocytes (43). The histological results of the current research showed higher hepatocyte death after heatstroke, which was alleviated to varying degrees by SePro, SePPs, PPs, and SeMet supplementation. This may be related to GSH content changes in the liver.

**FIGURE 7:**
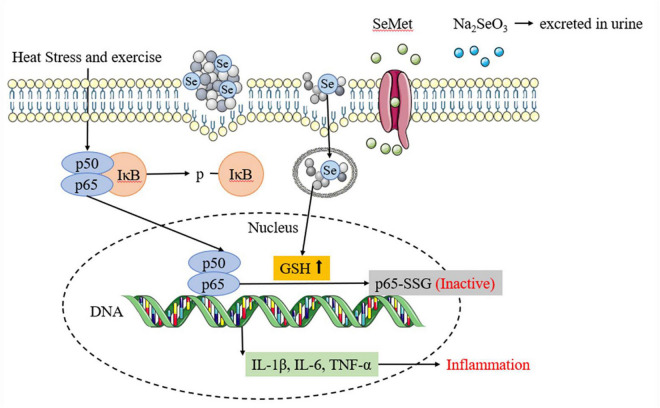
Different selenium supplements inhibits inflammatory response by affecting NF-κB/IκB signaling pathway.

The inflammatory response is another significant mechanism of heat stress and exercise fatigue-induced heatstroke (44). Selenium has been demonstrated to be able to attenuate the inflammatory response (45). In this study, SePPs inhibited the release of inflammatory factors (IL-1β, IL-6, and TNF-α) in the liver. The p-IκB and p65 protein expressions in the NF-κB/IκB signaling pathway were measured in the liver tissue to investigate the action mechanism of SePPs in suppressing the inflammatory response (see Figure 7). NF-κB is a nuclear transcription factor that also functions as an inflammatory mediator. The p50/p65 heterodimer is the common form of NF-κB present in hepatocytes and is inactivated by the binding of IκB to subunit p50/p65 in the cytoplasm (46). Xie et al. found that the NF-κB/IκB pathway plays a vital role in heat stress-induced ROS generation and cytotoxicity in the microvascular endothelial cells in rat lungs (47). In accordance to their results, heatstroke affected the NF-κB/IκB pathway and produced phosphorylated IκB, which was released from subunit p50/p65. It was ubiquitinated, converting subunit p50/p65 from an inhibited to an activated state, while activating the transcription of the gene-encoding proteins involved in the cellular response to stress factors (48). NF-κB controls pro-inflammatory cytokines and chemokines by increasing cell proliferation and stimulating angiogenesis. Consequently, limiting NF-κB activation potentially blocks cytokine expression, preventing the inflammatory response. Ge et al. reported that selenium supplementation attenuated Cd accumulation in the heart and reduced cardiac injury by affecting the NF-κB/IκB signaling pathway (49). In this investigation, supplementation with different forms of selenium and PPs before heat stress and exercise inhibited the p-IκB and p65 protein expression. Comparative analysis of the protective effects showed that SePPs inhibited the inflammatory response more effectively than other selenium supplements, indicating that selenium exerts an anti-inflammatory impact, while selenium and peptide binding is more easily absorbed and utilized by the body. SePPs could reduce inflammation by inhibiting the expressions of p-IκB and p65 proteins in the NF-κB signaling pathway. The DNA-binding activity of NF-κB can be regulated by redox, while the oxidation of its cysteine residues is necessary for the initiation of downstream effector proteins (50). The selenium and GSH Sytems display a unique relationship, rendering the liver GSH and GSH-Px significantly higher in the SePPs group than in the other groups. GSH can be engaged in various cellular reactions and can directly or indirectly modify the redox state of the liver *via* enzymatic reactions to scavenge free radicals and other reactive oxygen species (51). Furthermore, GSH also functions as a modifier of redox-sensitive proteins, especially since the protein contains cysteine and has a low pKa value. GSH can form mixed disulfides with cysteine in target proteins, a process known as glutathionylation (52). It is considered as a redox-dependent modification with potential relevance to signal transduction, metabolism, inflammation, and apoptosis, suggesting that GSH is an essential antioxidant and a crucial signaling molecule. Zhou et al. showed that GSH supplementation inhibited FKB-induced hepatocyte death *via* NF-κB and MAPK signaling (53). Proteomic studies have identified many proteins regulated by glutathionylation, including actin, Protein Kinase (PK) A, PKC, and NF-κB (54). NF-κB includes various subunits, of which p50 and p65 are the most abundant. The cysteine residue in the DNA-binding domain of the NF-κB p50 subunit can be glutathione, resulting in activity loss (55). The p65 subunit can also be modified by glutathionylation, causing a loss in activity (56). Since only p65 NF-κB is responsible for transcriptional activation (57), the effect of glutathionylation of the p65 subunit should have a stronger impact on NF-κB activation than that of the p50 subunit. These findings suggest that internal cysteine glutathionylation may be an important mechanism for protein expression regulation. Therefore, the reduced p65 protein level may also be due to the elevated GSH level leading to p65 glutathionylation. This protects sensitive cysteine residues from oxidation, restores protein function in oxidative stress conditions, and inhibits NF-κB activation, consequently reducing the degree of inflammation.

## Conclusion

This study provides *in vivo* evidence that SePPs protect rats from heat stress and exercise fatigue-induced heat stroke. SePPs improve the antioxidant capacity and inflammatory response of the liver in rats caused by heatstroke and reduce liver damage by inhibiting p-IκB and p65 protein expressions in the NF-κB/IκB pathway. It suggests that NF-κB/IκB may serve as a target for heatstroke treatment. In terms of boosting the GSH system, SePPs have a more significant impact than PPs. A comparison between the different selenium supplements revealed that combining selenium and peptides was more successful *in vivo*, suggesting that SePPs can be used as a preferential selenium resource. Alternatively, our study illustrates the potential of SePPs to prevent heatstroke and mitigate sports injury. Future research can focus on the structural interactions and metabolic pathways of SePPs to thoroughly understand their mode of action.

## Data Availability Statement

The raw data supporting the conclusions of this article will be made available by the authors, without undue reservation.

## Ethics Statement

The animal study was reviewed and approved by Institutional Animal Care and Use Committee at the Pony Testing International Group Co., Ltd. (PONY-2021-FL-15).

## Author Contributions

XC designed the research. XC and WL carried out experiments. XC and JZ analyzed the data. XC and HL wrote the initial manuscript, with contributions from YX and XL. All authors contributed to the article and approved the submitted version.

## Conflict of Interest

The authors declare that the research was conducted in the absence of any commercial or financial relationships that could be construed as a potential conflict of interest.

## Publisher’s Note

All claims expressed in this article are solely those of the authors and do not necessarily represent those of their affiliated organizations, or those of the publisher, the editors and the reviewers. Any product that may be evaluated in this article, or claim that may be made by its manufacturer, is not guaranteed or endorsed by the publisher.
